# Health-Related Quality of Life in Weight Loss Interventions: Results from the OPTIWIN Trial

**DOI:** 10.3390/ijerph18041785

**Published:** 2021-02-12

**Authors:** Livia Dainelli, Dan Roberto Luo, Sarah S. Cohen, Agnieszka Marczewska, Jamy D. Ard, Sally L. Coburn, Kristina H. Lewis, Judy Loper, Laura E. Matarese, Walter J. Pories, Amy E. Rothberg

**Affiliations:** 1 Nestlé Research, Nestlé, 1000 Lausanne, Switzerland; Livia.Dainelli@rdls.nestle.com (L.D.); DanRoberto.Luo@rd.nestle.com (D.R.L.); 2 EpidStrategies, Durham, NC 27101, USA; scohen@epidstrategies.com; 3 Nestlé Health Science, Nestlé, 1015 Lausanne, Switzerland; Agnieszka.Marczewska@nestle.com; 4 Department of Epidemiology and Prevention, Wake Forest School of Medicine, Winston-Salem, NC 27101, USA; jard@wakehealth.edu (J.D.A.); khlewis@wakehealth.edu (K.H.L.); 5 Alaska Premier Health, Anchorage, AK 99503, USA; chc2@mtaonline.net; 6 The Central Ohio Nutrition Center, Inc., Gohanna, OH 43230, USA; jloper@conci.com; 7 Department of Surgery, Brody School of Medicine, East Carolina University, Greenville, NC 27101, USA; mataresel@ecu.edu (L.E.M.); PORIESW@ecu.edu (W.J.P.); 8 Department of Nutritional Sciences, School of Public Health and Department of Internal Medicine, Michigan Medicine, University of Michigan, Ann Harbour, MI 48109-2029, USA

**Keywords:** obesity, health-related quality-of-life, weight loss interventions, IWQOL-Lite, WPAI-GH

## Abstract

Obesity is highly prevalent and associated with several adverse outcomes including health-related quality-of-life (HRQoL), work productivity, and activity impairment. The objective of this study is to examine group differences in HRQoL and labor-related health outcomes among participants in the OPTIWIN program, which compared the effectiveness of two intensive behavioral weight loss interventions. Participants (*n* = 273) were randomized to OPTIFAST^®^(OP) or food-based (FB) dietary interventions for 52 weeks. HRQoL and labor-related health outcomes were measured at baseline, week 26, and week 52, using two questionnaires. At baseline, there were no differences between groups on the Impact of Weight on Quality-of-Life Questionnaire (IWQOL-Lite). At week 26, the OP group had statistically significant differences towards better HRQoL for Physical Function, Self-Esteem, and the total score compared with the FB group. At week 52, the OP group showed better HRQoL in the total score (*p* = 0.0012) and in all but one domain. Moreover, the adjusted change-from-baseline normalized total score at week 52 was −5.9 points (*p* = 0.0001). Finally, the mean IWQOL-Lite normalized score showed that HRQoL improves by 0.4442 units (*p* < 0.0001) per kg lost, and that greater weight reduction was positively associated with better HRQoL. No statistically significant group differences were found with the Work Productivity and Activity Impairment (General Health) (WPAI-GH) Questionnaire. HRQoL improves with highly intensive, well-structured weight loss interventions. Greater weight loss lead to larger improvements. The lack of negative effect on productivity and activity suggests that these interventions may be compatible with an active work lifestyle.

## 1. Introduction

In the past four decades, the prevalence of obesity, defined as body mass index (BMI) of ≥ 30 kg/m^2^ [1], has been increasing in the US among each age, sex, and ethnic group [2], posing serious economic, human, and societal losses. Weight loss interventions, based on clinical (e.g., bariatric surgery and medications) and behavioral (e.g., diet and exercise) interventions, have been shown to impact not only physical health outcomes, but also health-related quality-of-life (HRQoL).

A systematic review of dietary interventions and health-related quality-of-life recognized a lack of data to support whether implementing dietary change positively or negatively affects HRQoL [3]. Several other studies have demonstrated the negative relationship between BMI and HRQoL, with greater impairments associated with extreme forms of obesity [4,5,6,7,8], and one study explored the amount of weight loss required to achieve clinically significant improvements in HRQoL [9]. 

The aim of this paper is to examine the tertiary outcomes of the OPTIWIN program, by comparing changes in HRQoL, work productivity, and activity impairment relative to weight loss among participants randomized into one of two intensive behavioral weight-loss interventions.

## 2. Materials and Methods

OPTIWIN (“Effectiveness of the Optifast Program Compared with a Reduced-energy Food-Based Diet Plan on Body Weight”) was an open-label, multicenter, randomized controlled clinical trial conducted over 52 weeks including a 26-week weight loss phase and a 26-week weight maintenance phase. The study was conducted at nine centers across the US comprised of both academic centers and freestanding weight loss clinics. After screening 463 participants (adult males and females, aged 18–70, non-smokers, with a BMI of 30–55 kg/m2) and confirming eligibility of 330 participants, 273 met eligibility criteria and were subsequently randomized and comprised the modified intention to treat (mITT) population (135 in the intervention and 138 in the comparator group) [10,11]. Retention rate remained high throughout the 52 weeks of the program. The intervention, referred to as “OP”, consisted of an OPTIFAST^®^ (Nestlé HealthCare Nutrition USA) program of total meal replacement that provided 800–1000 kcal/day depending on baseline BMI. The comparator, referred to as “FB”, consisted of a food-based reduced-energy diet plan (modified Diabetes Prevention Program diet) that historically focused on low fat (<30% of energy derived from fat), but modified to conform to the MyPlate which incorporates ~¼ plate of lean protein, ¼ of the plate of whole grains, and ½ plate of non-starchy vegetables. Participants were asked to log their food and track their calories in diaries which were reviewed weekly by the dietitian at which time further recommendations, if needed, were provided. Both treatments were combined with a behavioral component that included education, advice, and counseling about lifestyle change including getting 150–180 min of physical activity per week. The OP group demonstrated statistically significant greater weight loss compared to the FB group: at 52 weeks, weight loss was 10.5% (±0.6%) in the OP group versus 5.5% (±0.6%) in the FB group (*p* < 0.001). Details of the respective interventions, demographics, clinical characteristics, and outcomes have been published in detail elsewhere (Ard et al., Obesity 2019, Ard et al., Obesity Science & Practice, 2020, and Rothberg et al., Diabetes (supp) 2018).

In this study, we focused on the tertiary outcomes related to HRQoL and health-related labor outcomes using two validated instruments: Impact of Weight on Quality-of-Life (IWQOL-Lite) Questionnaire and Work Productivity and Activity Impairment (General Health) (WPAI-GH) Questionnaire. 

The IWQOL-Lite consists of 31 questions in 5 domains (Physical function, Self-esteem, Sexual life, Public distress, and Work). The normalized scores obtained range from 0 to 100 with higher scores implying better HRQoL [12].

The WPAI-GH consists of 6 questions and measures the impairment in work and regular activities during the past seven days. Three of the four subscores for WPAI-GH are about QoL in the workplace measuring Absenteeism, Presenteeism, and Work Productivity Loss, and thus were only scored among the subject currently employed. The scoring of the WPAI-GH produces impairment scores with a range of 0 to 100. Higher values indicate greater impairment and less productivity [13].

After examining univariate statistics for each measure, we developed multivariate linear regression models to assess potential differences between the OP and the FB groups for absolute values as well as change-from-baseline values for the IWQOL-Lite and the WPAI-GH. Models with change from baseline values included adjustment for baseline value. All models used the same covariates as in other OPTIWIN analyses [10,11], including age, sex, race/ethnicity, site, visit, and treatment and a treatment-by-visit interaction, unless differently specified. Baseline BMI was also included as a covariate in the models for IWQOL-Lite. Distributions of each of the absolute scores in the WPAI-GH were quite skewed with large proportions of respondents having a score of 0 (indicating no impairment).

## 3. Results

Table 1 shows the demographics of the modified intention to treat (mITT) population.

### 3.1. IWQOL-Lite

Table 2 provides a summary of normalized scores and descriptive statistics for each of the IWQOL-Lite domain by treatment group (OP vs. FB) at study baseline, at week 26, and at week 52.

Model results are shown in Table 3 and Table 4.

In Table 3, the normalized scores for absolute values are represented. At baseline, there was no difference in the normalized scores for the IWQOL-Lite (domains or total) between the OP and the FB group. At week 26, there were statistically significant improvements in the domains of Physical Functioning and Self-esteem in the OP group compared to the FB group. A non-statistically significant (*p* = 0.1556) improvement in the OP group was found in Sexual Life with normalized scores increasing from 73.3 to 87.4, in Public Distress (*p* = 0.0615) with normalized scores increasing from 81 to 90.7 and in Work (*p* = 0.2033) with normalized scores increasing from 84.3 to 91.4. More importantly, normalized overall total score LSM difference was 5.7 points higher in the OP group compared to the FB group (*p* = 0.0019), representing a significant improvement in HRQoL in the OP group after 26 weeks of treatment. Further improvements were experienced in week 52: the difference between groups turned significant in 4 out of 5 domains. Sexual Life improved from baseline to week 52 within each group, but the difference between groups remained not significant (*p* = 0.2415). The normalized overall total score LSM difference remained 6.1 points higher in the OP group compared to the FB group (*p* = 0.0012), implying that the significant improvement in HRQoL in the OP group, already witnessed at 26 weeks, persisted to 52 weeks at study completion.

Similar results were found for the change-from-baseline for the normalized IWQOL-Lite scores (Table 4). In week 26, the LSM Difference between OP and FB reached significance for 3 out of 5 of the domains and for the overall total with 5.5 points difference (*p* = 0.0001). Self-Esteem was the most positively impacted domain with an improvement of over 7.6 points (*p* = 0.0014). At week 52, all the domains, excluding Sexual life (*p* = 0.2706), reached significance and the Overall Total remained significant with 5.8 points difference (*p* = 0.0001).

Additionally, the Pearson correlation between IWQOL-Lite overall total score change-from-baseline and weight reduction from baseline at week 26, 0.42033 (*p* < 0.0001), and at week 52, 0.46138 (*p* < 0.0001), suggest that greater weight reduction is positively associated with better HRQoL.

Finally, we analyzed the variation in the change-from-baseline IWQOL-Lite normalized score per unit of weight loss (in kg) from linear mixed models. In addition to the previously used covariates (age, sex, race/ethnicity, site, visit, baseline BMI, treatment, and a treatment-by-visit interaction), for this specific analysis, we included weight reduction, baseline IWQOL-Lite score, and weight reduction-by-treatment interaction term. Based on a multivariate model, the mean IWQOL-Lite normalized score on the combined sample of OP and FB increased 0.4442 (95% CI, 0.3266–0.5617) per kg lost, again underscoring a strong positive relationship between weight-loss and HRQoL.

### 3.2. WPAI-GH

Table 5 provides descriptive statistics for the WPAI-GH subscores by treatment group (OP vs. FB) at study baseline, at week 26, and at week 52.

For the WPAI-GH subscores, LSM differences for change-from-baseline values in week 26 were not statistically significant (Table 6). The same pattern remained at week 52, suggesting that there was no increase in worker absenteeism or decrement in work productivity. The LSM change-from-baseline WPAI-GH sub-scores for Absenteeism, Presenteeism, and Work productivity loss within the OP and FB groups did show change in the direction of better QoL. Activity impairment, in particular, showed a reduction of 6.2 points compared to baseline at week 52 in the OP group.

## 4. Discussion

In the OPTIWIN study, a randomized, controlled clinical trial comparing two intensive behavioral weight loss interventions, HRQoL, work productivity, and activity impairment were assessed using two validated instruments: the IWQOL-Lite and WPAI-GH. Findings from our analyses support the evidence that greater weight loss has greater impact on HRQoL. The OP intervention resulted in significantly greater weight loss compared to the FB intervention, and as a result, improved HRQoL to a greater extent.

The IWQOL-Lite showed statistically significant differences both in absolute and change-from-baseline values between the OP and FB groups. With the exception of Sexual Life, there were statistically significant improvements in all other domains and the overall total score, with greater improvements in the OP group suggesting that the greater reduction in weight translated into greater improvements in HRQoL. Although the difference between groups in Sexual life was not significant, increases in score were seen within groups: normalized scores increased from 73.3 at baseline to 86.9 at week 52 in the OP group and from 72.1 to 83.2 in the FB group, consistent with the literature showing that overweight/obese individuals who lose weight improve their sexual life over time [14,15]. Previous literature suggests that weight loss ≥10% (10.5% in the OP group) is needed by people with severe obesity to achieve minimal clinically important differences in HRQoL [16]. Therefore, clinicians should not just settle for minimal weight loss if more effective options are available.

Results from WPAI-GH showed no statistically significant difference in work productivity and activity impairments between the OP and the FB group. This could be due to a ceiling effect, i.e., a large proportion of respondents had a score of 0, indicating no impairment at the start and hence no room for improvement. Indeed, given the substantial time commitment that individuals devoted to the program (travel to and from the clinic site, time at site visit, in-person weekly counseling sessions, and the requirement for 150–180 min of physical activity per week), it is notable that participants did not experience significant worsening in absenteeism or presenteeism and maintained the same level of productivity at work. These findings indicate that even highly intensive behavioral weight loss interventions are compatible with an active work lifestyle which is consistent with other reports [17].

There is a vast literature of published trials reporting the effects of intensive behavioral weight loss interventions on HRQoL, work productivity, and activity impairment. Their results go in the same direction of ours (improved HRQoL and labor outcomes after weight loss) even when accounting for differences (e.g., length of trial, treatment, instruments used, and mITT population characteristics) among studies [18,19,20,21].

Imayama et al. compared the effects of diet and exercise interventions on health-related quality-of-life (measured by the SF-36) and psychosocial conditions, such as stress (Perceived Stress Scale), depression (Brief Symptom Inventory (BSI)-18), anxiety (BSI-18), and social support (Medical Outcome Study Social Support Survey) [18]. Four-hundred-and-thirty-nine postmenopausal women were randomly allocated to diet, exercise, diet, and exercise combined or to a control group. They found that the combined intervention of diet and exercise led to a more favorable impact on HRQoL and psychological health compared to diet or exercise alone.

Pearl et al. compared the effects of weight loss and weight loss maintenance on specific health-related quality-of-life measures including the IWQOL-Lite, the Patient Health Questionnaire-9 (depression), and the Perceived Stress Scale [19]. Adult patients (*n* = 137) with obesity participated in a 14-week intensive lifestyle intervention/low-calorie diet program. Those who lost ≥ 5% of initial weight were randomly assigned to lorcaserin, a weight loss drug (now withdrawn from the US market), or placebo for an additional 52 weeks. Except for weight-related public distress, significant improvements in all outcomes measured on the IWQOL-Lite were found during the initial 14 weeks and largely maintained during the subsequent 52 weeks.

Kaukua et al. randomized 38 obese men (BMI ≥35 kg/m^2^) to a very low energy diet or to a control group and measured HRQoL using the RAND 36-Item Health Survey 1.0 and obesity-related psychosocial problems scale [20]. While the weight in the control group remained stable, the intervention group lost 17% at the end of the first 4-month program and 13.9% at the end of the 4-month maintenance phase. Their findings were also in keeping with the other studies and showed that significant weight loss leads to improvements in physical functioning, social functioning, obesity-related psychosocial problems, and perceived health.

Finally, Rothberg et al. studied HRQoL using the EQ-5D index and VAS in 188 patients with severe obesity (mean BMI 40 kg/m^2^) and co-morbid health conditions. The authors showed that “measured improvements in HRQoL between baseline and follow-up were greater than predicted by the reduction in BMI at follow-up” and “and that the improvement in HRQL for each kilogram lost or percentage of body weight lost is greater than would be predicted by assessing the cross-sectional relationship between BMI and HRQoL”. They also indicated that patients tend to underestimate the impact that weight has on HRQoL, and therefore measuring it before and after weight loss is paramount to understanding the relationship of weight to HRQoL [21]. Based on the aforementioned studies, the evidence suggests that effective weight loss interventions improve HRQoL and seems to not deteriorate work productivity and activity impairments. However, more straightforward comparisons between OPTWIN and other trials are challenging due to differences in population, strategies, duration of interventions, and HRQoL measurement instruments used. Overall, the promising results from this study, confirm that comprehensive, behavioral weight loss interventions have a positive impact on HRQoL and have not significant effect in work productivity and activity impairment.

The strengths of this study are related to the strength of the OPTIWIN trial, including a study design with active comparator, multiple sites across the US, the large sample size, and the high retention rate. In addition, both diets were paired with behavioral support, education and counseling, a necessary component to promote long-term lifestyle modifications, and durable weight loss.

Limitations of the study include the composition of the sample which was predominantly female, and therefore limits generalizability to the overall population with obesity, and that measuring health-related quality-of-life questionnaires was part of the trial’s tertiary objective, and therefore the study may not have been powered to detect differences of the magnitude observed here. However, the latter could help to explain some of the non-significant results. Finally, concerns exist about the sustainability of the weight loss in the long-term as what happens months or years after the initial weight loss depends on the individual behavior and not only on the goodness of the approach followed to lose weight. Nevertheless, there is literature demonstrating that meal replacements are a sustainable weight loss approach as, in the long-term (1 to 4 years), they help to lose more weight than other interventions and facilitate weight maintenance [22,23].

## 5. Conclusions

The analysis of tertiary outcomes of the OPTIWIN program suggests that HRQoL improves with highly intensive and carefully designed interventions that help individuals with obesity to lose weight. The greater the weight reduction the better HRQoL. The lack of impact on worker productivity suggests that these interventions are compatible with an active work lifestyle.

## Figures and Tables

**Table 1:** Summary of subject demographics modified intention to treat (mITT) population.

	OPTIFAST Program (N = 135)	Food-Based Program (N = 138)	Overall (N = 273)
**Age (years)**			
** Mean (SD)**	47.0 (11.2)	47.2 (11.3)	47.1 (11.2)
** Range**	20, 66	21, 70	20, 70
**Sex, n (%)**			
** Male**	19 (14.1)	29 (21.0)	48 (17.6)
** Female**	116 (85.9)	109 (79.0)	225 (82.4)
**Race, n (%)**			
** Caucasian**	100 (74.1)	95 (68.8)	195 (71.4)
** African American**	22 (16.3)	37 (26.8)	59 (21.6)
** Asian/Pacific Islander**	4 (3.0)	2 (1.4)	6 (2.2)
** Hispanic**	5 (3.7)	4 (2.9)	9 (3.3)
** Native American**	1 (0.7)	0	1 (0.4)
**Other** [1]	3 (2.2)	0	3 (1.1)
**Educational Level, n (%)**			
** Some High School**	1 (0.7)	0	1 (0.4)
** High School/GED**	34 (25.2)	28 (20.3)	62 (22.7)
** Professional Degree**	8 (5.9)	17 (12.3)	25 (9.2)
** Undergraduate Degree**	53 (39.3)	51 (37.0)	104 (38.1)
** Masters**	28 (20.7)	23 (16.7)	51 (18.7)
** Doctoral**	6 (4.4)	7 (5.1)	13 (4.8)
** Some Graduate Work**	4 (3.0)	6 (4.3)	10 (3.7)
** No Response**	1 (0.7)	6 (4.3)	7 (2.6)
**Household Income Level, n (%)**			
** Less Than $24,999**	15 (11.1)	18 (13.0)	33 (12.1)
** $25,000 to $49,999**	27 (20.0)	33 (23.9)	60 (22.0)
** $50,000 to $99,999**	56 (41.5)	50 (36.2)	106 (38.8)
** $100,000 or more**	22 (16.3)	27 (19.6)	49 (17.9)
** No Response**	15 (11.1)	10 (7.2)	25 (9.2)

**Table 2:** Summary of IWQOL-Lite Normalized Scores by Visit in the mITT Population (Observed Cases).

	Baseline		Visit 3/Week 26		Visit 5/Week 52	
	OPTIFAST Program	Food-Based Program	OPTIFAST Program	Food-Based Program	OPTIFAST Program	Food-Based Program
**Physical Function**						
N	135	137	116	121	112	108
Mean (SD)	68.3 (20.36)	69.8 (19.17)	85.7 (14.05)	79.7 (18.44)	85.7 (15.34)	80.1 (18.36)
Min, Max	18, 100	16, 100	34, 100	23, 100	25, 100	14, 100
**Self-Esteem**						
N	135	137	116	121	112	108
Mean (SD)	55.6 (24.37)	57.6 (25.66)	75.8 (20.85)	68.1 (26.01)	75.7 (22.55)	68.7 (26.21)
Min, Max	0, 100	0, 100	21, 100	0, 100	4, 100	0, 100
**Sexual Life**						
N	131	130	109	115	110	106
Mean (SD)	73.3 (27.15)	72.1 (26.24)	87.4 (17.35)	82.5 (19.54)	86.9 (22.08)	83.2 (21.14)
Min, Max	0, 100	0, 100	19, 100	19, 100	0, 100	0, 100
**Public Distress**						
N	135	135	114	118	112	107
Mean (SD)	81.0 (21.41)	81.3 (22.06)	90.7 (15.00)	86.1 (19.11)	91.7 (13.96)	88.6 (17.56)
Min, Max	15, 100	15, 100	25, 100	30, 100	40, 100	10, 100
**Work**						
N	131	133	113	115	111	107
Mean (SD)	84.3 (18.92)	85.5 (17.97)	91.4 (16.16)	89.7 (16.60)	94.2 (11.39)	90.2 (15.69)
Min, Max	25, 100	31, 100	31, 100	25, 100	50, 100	31, 100
**Overall Total**						
N	134	135	113	118	111	107
Mean (SD)	70.1 (17.46)	71.2 (17.53)	85.3 (13.16)	79.7 (17.01)	85.5 (13.97)	80.5 (16.64)
Min, Max	18, 100	28, 100	42, 100	32, 100	38, 100	35, 100

Note: IWQOL = Impact of Weight on Quality of Life; SD = Standard Deviation. Normalized Score is converted from Raw Score on a scale of 0–100.

**Table 3:** Linear mixed models results for IWQOL-Lite normalized scores ^a^ absolute values, mITT population.

	OPTIFAST			Food-Based			LSM Difference ^b^	*p-Value* ^c^
	N	LSM	SE	N	LSM	SE	(95% CI)	
**Baseline**								
Physical Function	135	68.1381	1.3570	137	69.6861	1.3412	1.5479 (−2.2316, 5.3275)	0.4213
Self-Esteem	135	56.4817	1.9686	137	56.9776	1.9450	0.4959 (−4.9880, 5.9798)	0.8590
Sexual Life	131	73.6398	1.9684	130	71.7920	1.9559	−1.8478 (−7.3455, 3.6498)	0.5092
Public Distress	135	80.7579	1.2757	135	81.7032	1.2686	0.9453 (−2.6175, 4.5081)	0.6023
Work	131	84.4903	1.3599	133	85.2371	1.3463	0.7469 (−3.0456, 4.5393)	0.6989
Overall Total	134	70.1390	1.2243	135	70.9800	1.2124	0.8410 (−2.5743, 4.2563)	0.6287
**Week 26**								
Physical Function	116	85.9340	1.4247	121	79.8138	1.3987	−6.1201 (−10.0742, −2.1661)	0.0025
Self-Esteem	116	76.5103	2.0583	121	67.6352	2.0214	−8.8751 (−14.5905, −3.1596)	0.0024
Sexual Life	109	86.8678	2.0906	115	82.6809	2.0415	−4.1869 (−9.9724, 1.5985)	0.1556
Public Distress	114	90.7165	1.3562	118	87.1249	1.3348	−3.5916 (−7.3576, 0.1744)	0.0615
Work	113	91.9258	1.4330	115	89.3360	1.4208	−2.5898 (−6.5848, 1.4052)	0.2033
Overall Total	113	85.4996	1.2886	118	79.8068	1.2649	−5.6928 (−9.2699, −2.1157)	0.0019
**Week 52**								
Physical Function	112	85.7271	1.4397	108	79.8229	1.4497	−5.9042 (−9.9488, −1.8596)	0.0043
Self-Esteem	112	76.9563	2.0781	108	67.9186	2.0885	−9.0377 (−14.8722, −3.2032)	0.0025
Sexual Life	110	87.7738	2.0830	106	84.2828	2.0971	−3.4910 (−9.3410, 2.3590)	0.2415
Public Distress	112	92.3008	1.3652	107	87.9218	1.3836	−4.3790 (−8.2264, −0.5316)	0.0258
Work	111	95.1552	1.4417	107	89.8243	1.4589	−5.3309 (−9.3923, −1.2695)	0.0102
Overall Total	111	86.2735	1.2950	107	80.2228	1.3030	−6.0507 (−9.6898, −2.4116)	0.0012

^a^ Normalized score values have range of 0 to 100 with higher scores representing better QOL. ^b^ LSM Difference = Least squares mean difference [Food-based LSM-OPTIFAST LSM]. ^c^ *p*-value compares OPTIFAST to Food-based program.

**Table 4:** Linear mixed models result for IWQOL-Lite normalized score ^a^ change-from-baseline values, mITT population.

	OPTIFAST			Food-Based			LSM Difference ^b^	*p-Value* ^c^
	N	LSM	SE	N	LSM	SE	(95% CI)	
**Week 26**								
Physical Function	116	17.0033	1.1136	120	10.9685	1.0996	−6.0348 (−9.1521, −2.9175)	0.0002
Self-Esteem	116	19.2701	1.6514	120	11.6687	1.6289	−7.6015 (−12.2190, −2.9840)	0.0014
Sexual Life	108	14.0972	1.5478	108	11.0814	1.5495	−3.0159 (−7.3768, 1.3451)	0.1741
Public Distress	114	9.9954	1.0528	115	5.8540	1.0504	−4.1414 (−7.1014, −1.1814)	0.0063
Work	110	7.3046	1.1810	111	4.6960	1.1775	−2.6086 (−5.9360, 0.7189)	0.1237
Overall Total	113	14.9704	0.9776	115	9.4770	0.9713	−5.4934 (−8.2397, −2.7470)	0.0001
**Week 52**								
Physical Function	112	16.9693	1.1253	107	10.9787	1.1402	−5.9907 (−9.1791, −2.8022)	0.0003
Self-Esteem	112	19.9098	1.6693	107	12.1313	1.6920	−7.7785 (−12.5083, −3.0487)	0.0014
Sexual Life	108	14.7212	1.5490	99	12.2200	1.6160	−2.5012 (−6.9671, 1.9647)	0.2706
Public Distress	112	11.4335	1.0619	105	6.9273	1.0928	−4.5062 (−7.5406, −1.4717)	0.0038
Work	107	10.2611	1.1957	104	5.3818	1.2106	−4.8793 (−8.2769, −1.4818)	0.0051
Overall Total	110	15.8234	0.9878	105	9.9670	1.0044	−5.8564 (−8.6654, −3.0474)	0.0001

^a^ Normalized score values have range of 0 to 100 with higher scores representing better QOL. ^b^ LSM Difference = Least squares mean difference [Food-based LSM-OPTIFAST LSM]. ^c^ *p*-value compares OPTIFAST to Food-based program.

**Table 5:** Summary of WPAI-GH Subscores by Visit in the mITT Population (Observed Cases).

	Baseline		Visit 3/Week 26		Visit 5/Week 52	
	OPTIFAST Program (N = 135)	Food-Based Program (N = 138)	OPTIFAST Program (N = 135)	Food-Based Program (N = 138)	OPTIFAST Program (N = 135)	Food-Based Program (N = 138)
**Absenteeism**						
** N**	108	102	91	98	90	87
** Mean (SD)**	1.8 (6.64)	0.9 (4.53)	1.4 (6.42)	2.8 (14.82)	3.7 (17.09)	0.9 (3.31)
** Min, Max**	0, 50	0, 40	0, 50	0, 100	0, 100	0, 17
**Activity Impairment**						
** N**	131	135	114	119	112	107
** Mean (SD)**	18.1 (24.90)	15.4 (23.20)	12.1 (21.92)	11.8 (22.70)	9.6 (17.11)	10.6 (19.07)
** Min, Max**	0, 90	0, 100	0, 80	0, 90	0, 80	0, 80
**Presenteeism**						
** N**	106	101	94	98	86	87
** Mean (SD)**	10.8 (18.50)	7.3 (15.36)	5.4 (12.33)	7.3 (19.77)	5.8 (12.78)	6.8 (14.26)
** Min, Max**	0, 90	0, 100	0, 60	0, 100	0, 60	0, 60
**Work productivity**						
** N**	106	101	90	98	85	87
** Mean (SD)**	11.8 (20.10)	7.6 (15.60)	6.4 (14.41)	7.8 (20.23)	6.4 (13.78)	7.5 (15.04)
** Min, Max**	0, 92	0, 100	0, 70	0, 100	0, 68	0, 64

Note: WPAI-GH = Work Productivity and Activity Impairment for General Health. The questions are based on the past 7 days.

**Table 6:** Linear mixed models results for WPAI-GH change-from-baseline values, mITT population.

	OPTIFAST			Food-Based			LSM Difference ^b^	*p-Value* ^c^
	N	LSM	SE	N	N	LSM	(95% CI)	
**Week 26**								
**Absenteeism**	83	−0.0841	1.4130	85	1.7346	1.3967	1.8187 (−2.1577, 5.7951)	0.3674
**Presenteeism**	84	−2.8152	1.6347	84	−0.1154	1.6376	2.6998 (−1.9373, 7.3370)	0.2516
**Work productivity**	80	−2.5220	1.7781	84	−0.1316	1.7433	2.3904 (−2.6039, 7.3847)	0.3455
**Activity Impairment**	111	−4.6041	1.7381	117	−2.4245	1.6904	2.1796 (−2.6543, 7.0135)	0.3750
**Week 52**								
**Absenteeism**	83	2.3464	1.4111	77	−0.1404	1.4629	−2.4867 (−6.5498, 1.5764)	0.2283
**Presenteeism**	78	−3.2866	1.6858	76	−0.8245	1.6982	2.4622 (−2.3387, 7.2631)	0.3123
**Work productivity**	77	−2.9930	1.8074	76	−0.4983	1.8071	2.4947 (−2.6337, 7.6230)	0.3377
**Activity Impairment**	108	−6.1762	1.7580	105	−3.2009	1.7749	2.9753 (−2.0075, 7.9581)	0.2404

Models only included subjects who answered “Yes” to “Are you currently working?” ^b^ LSM Difference = Least squares mean difference [Food-based LSM-OPTIFAST LSM]. ^c^ *p*-value compares OPTIFAST to Food-based program.

## Data Availability

Not applicable.

## Clinical Trial Registration

Clinicaltrials.gov identifier NCT02635698. Retrospectively registered on November 2015.
